# The efficacy of custom-made offloading devices for diabetic foot ulcer prevention: a systematic review

**DOI:** 10.1186/s13098-024-01392-y

**Published:** 2024-07-24

**Authors:** Aled Wyn Jones, Abraham Makanjuola, Nathan Bray, Yeliz Prior, Daniel Parker, Christopher Nester, Jinghua Tang, Liudi Jiang

**Affiliations:** 1 Academy for Health Equity, Prevention and Wellbeing, Bangor University, Bangor, UK; 2 Faculty of Life Sciences and Education, University of South Wales, Cardiff, UK; 3 Centre for Human Movement and Rehabilitation, School of Health and Society, University of Salford, Salford, UK; 4 School of Allied Health Professions, University of Keele, Keele, UK; 5 School of Engineering, Faculty of Engineering and Physical Sciences, University of Southampton, Southampton, UK

**Keywords:** Diabetes, Foot ulcer, Peripheral neuropathy, Offloading devices, Insoles, Orthotics

## Abstract

**Introduction:**

Therapeutic offloading devices, including insoles, shoes, and other orthoses, are some of the most commonly used interventions to treat or prevent diabetic foot ulceration. Custom-made offloading devices are increasingly used to offset the development of foot ulcers. However, whether these devices are more effective than prefabricated standard offloading devices is uncertain. Therefore, this systematic review collates and examines evidence on the efficacy of custom-made offloading devices in preventing foot ulcer incidence and recurrence in people diagnosed with diabetes.

**Methods:**

Five scientific databases were searched, covering 2011–2023. Initial searches and screening were carried out independently by two researchers. Studies meeting the inclusion criteria were further examined through additional screenings, and critical appraisal. Data relevant to the review aims were extracted and analysed within a narrative synthesis.

**Results:**

Of the 1,715 articles found in the initial searches, nine papers were found to meet inclusion criteria and were included in the review. The evidence shows that custom-made offloading devices are likely to be more effective for reducing or preventing diabetic foot ulcers than standard offloading devices. However, due to a lack of data it remains uncertain whether custom-made offloading devices are more cost-effective for preventing ulceration compared to standard insoles. Likewise, due to measurement heterogeneity between studies and lack of data, it is unclear whether adherence is higher in users of custom-made offloading devices, and whether such devices deliver significantly greater reductions in peak pressure as compared to standard offloading devices.

**Conclusion:**

Custom-made offloading devices are more effective than standard devices for preventing diabetic foot ulceration, and we recommended their use when feasible; however, there remains uncertainty regarding their cost-effectiveness compared to standard insoles and offloading devices.

## Background

Diabetes mellitus (hereafter referred to as diabetes) refers to a group of endocrinological diseases characterised by prolonged hyperglycaemia [1, 2]; type 2 diabetes is the most common form of diabetes, followed by type 1 diabetes. Though the pathophysiology of type 1 and type 2 diabetes differ significantly, both share similar complications if left untreated or poorly controlled [3–5]. Of these complications, diabetic foot ulceration (DFU) is among the most serious, incurring considerable costs for individuals, families, and health systems alike [6–9]. Characterised by the formation of deep, slow healing wounds on the lower limbs, DFU is the primary antecedent for lower limb amputation in diabetic patients [10, 11] which in turn leads to further burdens on patients and healthcare providers [12, 13].

The aetiology of DFU is multifactorial [14, 15]; among the most significant factors is peripheral neuropathy, a condition caused by damage to the peripheral nervous system from prolonged hyperglycaemia [16]. Long-term, this is the most common complication of diabetes [17] and includes symptoms such as sensory impairment, paraesthesia, and weakness in affected areas [18]. The loss of sensation is arguably the most consequential in terms of DFU development; those affected are unable to detect and avoid harmful stimuli such as shearing forces or accidental trauma, leading to skin damage and eventual ulcer formation [19, 20]. Preventative measures which reduce the incidence of DFU are paramount to improving the outcomes of people with diabetes.

Therapeutic offloading devices for the feet are commonly used to prevent the development of DFU. However, Paton et al. [21] found only limited evidence that standard generic insoles can reduce the incidence of ulceration in diabetic patients. The use of custom-made offloading devices preventing DFU incidence may offer more efficacy by accounting for multiple individual factors, and several studies examining variable offloading devices have so far demonstrated positive results [22–24].

Despite positive evidence, the overall clinical-effectiveness and cost-effectiveness of custom-made offloading devices for diabetic patients remains unclear. Healthcare systems have limited resources, thus it is important that clinical interventions result in significant health benefits at acceptable costs per patient. Therefore, the aim of this systematic review is to evaluate the effectiveness and cost-effectiveness of custom-made offloading devices as compared to standard offloading devices for the prevention of DFU.

## Methods

The protocol for this study was registered with International Prospective Register of Systematic Reviews (PROSPERO) under registration number CRD42023429948.

### Aim

The primary aim of this systematic review was to determine the efficacy of custom-made offloading devices and variable insoles for the prevention of DFU, as compared to standard offloading devices. The secondary aims were:

1. To determine which lower limb offloading devices provide the greatest risk reduction for ulceration.
2. To establish rates of adherence among users of custom-made devices and whether adherence reduces ulceration.
3. To determine whether variable/custom-made devices are more cost-effective than standard/generic devices for the prevention/treatment of DFU.

### Review methodology

A systematic review was conducted and reported according to the Cochrane Handbook for Systematic Reviews of Interventions [25].

### Data sources & search strategy

The electronic databases MEDLINE, Scopus, Embase, Web of Science, and Cochrane Library were systematically searched for potential research studies. Additionally, reference lists of publications were analysed to identify further potential candidate papers. Grey literature and thesis databases were not scanned due to time constraints. The publication time period was limited to 2011–2023 and only English language articles were included.

The search strategy initially consisted of broad-stroke terms, including “foot ulcer”, “insoles”, and “orthosis”. Subsequent analysis and extraction of keywords from the titles, abstracts, and index terms of retrieved articles were used to refine the final search strategy, as displayed in Table 1. Terms were decided by consulting with expert clinicians and researchers.

**Table 1:** Search terms and keywords

Field	Search terms	Boolean
1	“diabetic foot ulcer*” OR “foot ulcer*” OR “diabetic neuropath*” OR “diabet* complications” OR “neuropath* ulcer*”	AND
2	“insole*” OR “orthos*” OR “orthotic*” OR “shoe*” OR “footwear” OR “cast*”	AND
3	“incidence” OR “incidence ratio” OR “offload*” OR “risk” “OR “risk reduction” OR “cost*” OR “cost-effectiveness”	

Search results were downloaded from each database and saved as comma-delimited values. Primary screening of titles and abstracts was conducted independently by two investigators (AWJ and AM), with a third investigator on hand to act as an impartial third-party in cases of disagreement (NB). Secondary screening of full titles was conducted by two investigators (AWJ and AM) together. Inclusion and exclusion criteria are presented in Table 2.

**Table 2:** Inclusion and exclusion criteria for retrieved studies

	Inclusion criteria	Exclusion criteria
Participants	Adults formally diagnosed with type 1 or type 2 diabetes and with current or previous lower limb ulceration	People without either diabetes or DFU
Intervention/exposure	Insoles, orthoses, or other footwear designed to offload pressure and reduce foot ulceration incidence	Interventions not involving the use of insoles or other offloading devices
Comparison	Any randomised control trial, cohort study, or study design involving comparison of control groups to experimental groups	Case reports, case studies, or any other design that focuses on single cases of disease
Outcomes	Relative risk reduction, including measurements by standardised tools such as Wagner’s classification scale Incidence of foot ulceration, including incidence percentages and incidence risk ratios (IRRs) Health economic costs, including quality-adjusted life years (QALYs), unit and service costs, and estimated benefits	Lacking any of the outcomes for inclusion
Publication type	Published and preprint	Not peer-reviewed

### Quality assessment

To assess the methodological quality of studies included in the review, the critical assessment tools developed by JBI [26] were utilised. Briefly, these tools provide assessors with checklists to evaluate different aspects of studies, including their designs, measurements, and data analyses; assessors then score these evaluations and assign a quality rating of “low”, “medium”, or “high” depending on said score. Studies were assessed independently by two investigators (AWJ and AM), with a third party available to settle any disagreements in scoring and/or quality rating (NB).

### Data extraction

The following data were extracted from selected studies:

- Publication data: author(s), year.
- Methodology data: study design, inclusion criteria, outcome measurements, intervention description, setting.
- Statistical data: number of participants, baseline characteristics, statistical results.
- Text/other data: other results.

### Data analysis and synthesis

A narrative synthesis approach was employed to analyse and surmise data extracted from selected studies. The structure of the synthesis was based on the original aims, with the primary aim of determining whether custom-made offloading devices prevent ulcer recurrence/incidence, as compared to standard offloading devices. The secondary aims were evaluated in no particular order.

## Results

### Search results

**Fig. 1:**
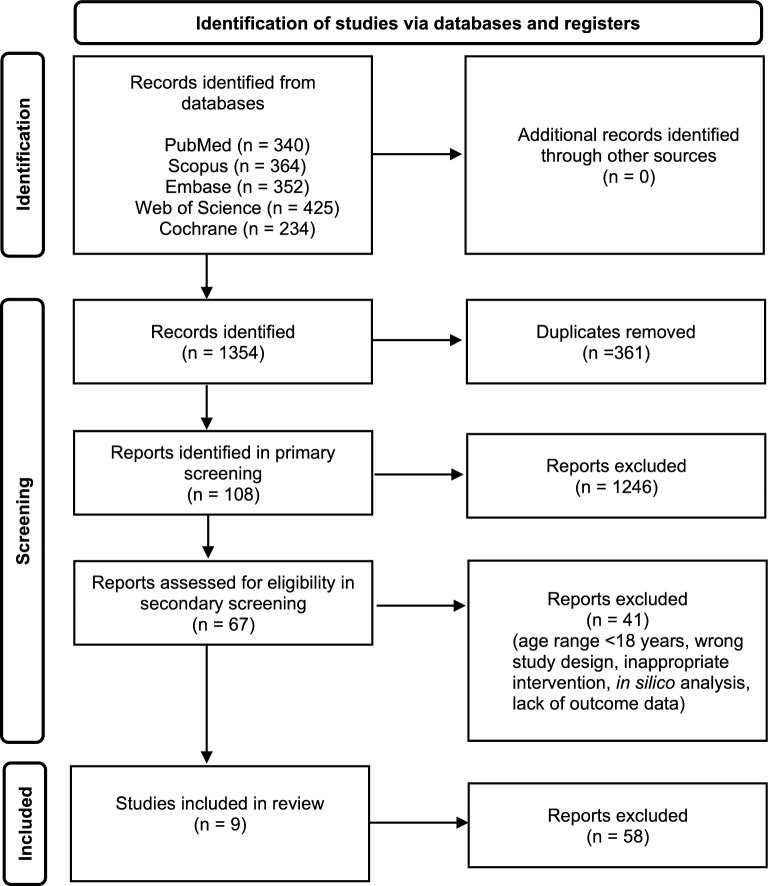
PRISMA diagram

A total of 1,715 articles were retrieved from across the five databases during the search stage; removal of duplicates reduced this number to 1,354. Primary screening extracted 108 articles for further analysis. At secondary screening, 67 studies were considered eligible for inclusion. Nine studies met inclusion criteria after review of full-texts, and were included in the final narrative synthesis. See Fig. 1 for full results.

### Details of selected studies

Extracted data with categorised information can be found in Table 3. Six papers were randomized controlled trials (RCTs) [21, 27–31]; one was an economic evaluation/model [32]; one was a non-controlled intervention study (interpreted as quasi-experimental for quality appraisal purposes) [33]; and one was a randomised multicentre prospective study (appraised as an RCT for ease of comparison) [34].

**Table 3:** Data extraction table for studies included in final narrative synthesis

Author(s) & year	Study design\setting	Inclusion criteria	No. of participants	Baseline characteristics*	Intervention(s)/follow up	Outcomes	Results
Collings et al. (2023) [27]	RCT with embedded qualitative study Three study sites across South West England	≥ 18 years old Diabetic (T1/T2) Sensory neuropathy	61 diabetic persons with neuropathy Control: 31 Intervention: 30	• Intervention: Sex (m/f): 27/3 Age (years): 70.2 ± 10.2 Diabetes type (T1/T2): 2/28 Duration of diabetes (years): 19.7 ± 14.9 Previous ulceration: 15 • Control: Sex (m/f): 26/5 Age (years): 67.9 ± 12.2 Diabetes type (T1/T2): 7/24 Duration of diabetes (years): 21.3 ± 9.7 Previous foot ulceration: 16	Instant insoles optimised by F-scan in-shoe pressure analysis system and treatment algorithm Follow-up: 3, 6 & 12 months	• Plantar foot ulceration incidence • Insole adherence as measured by temperature sensor • Nottingham Assessment of Functional Footcare and International Physical Activity questionnaires • Assessment of trial safety by adverse event data • Mean peak plantar pressure	1) Ulceration incidence (12-months): • Intervention: 33.3% (10/30) • Control: 22.5% (7/31) 2) Insole adherence rates: • < 4 h/day: 45.5% (20) • 4–8 h/day: 38.6% (17) • > 8 h/day: 15.9% (7) 3) Mean peak plantar pressure (all regions; adjust for baseline difference only): • 3 month follow-up: 77.3 (CI 61.5, 216.2) • 6 month follow-up: 44.5 (CI 148.5, 237.5) • 12 month follow-up: 239.4 (CI 35.4, 514.2) 4) Adverse Events (AE) and Severe Adverse Events (SAE): • Overall AE: 26 • Intervention: 17 AE • Control: 9 AE • Overall SAE: 6 • No SAE attributed to intervention
Keukenkamp et al. (2022) [33]	Quasi-experimental study (pre-post design) Three multidisciplinary diabetic foot outpatient clinics in the Netherlands	Diabetic (T1/T2) Moderate to high risk of ulceration	31 diabetics persons at risk of ulceration Baseline low indoor adherence: 23 Baseline high low adherence: 8	• Baseline low indoor adherence: Age (years): 68.3 ± 11.2 Female gender: 39% (9) BMI (kg/m^2^): 30 ± 7 Diabetes (T2): 78% (18) Diabetes duration (yrs): 19.5 ± 15.7 LOPS: 100% (23) • Baseline high indoor adherence: Age (years): 72.1 ± 4.2 Female gender: 50% (4) BMI (kg/m^2^): 32 ± 8 Diabetes (T2): 75% (6) Diabetes duration (yrs): 19.5 ± 10.2 LOPS: 100% (8)	Custom-made footwear for indoor use Follow-up: 1 & 12 months	• Footwear adherence • Mean peak plantar pressure • Ulcer recurrence rate	1) Footwear adherence: in low adherence. group: • Increased significantly from Median 65–77% (1 month; *p* = 0.002) and Median 87% (12 months; *p* < 0.001), • Time footwear worn increased significantly from 8.6 h/day to 9.3 h/day (1 month; *p* = 0.0014, *r* = 0.68) and 12.0 h/day (12 months; *p* = 0.002, *r* = 0.75) 2) Mean peak plantar pressure: • Only one significant difference found: right heel region (Indoor footwear: 185 ± 58 kPa, regular footwear: 209 ± 69 kPa; mean difference: − 24 (− 47 to − 1); *p* = 0.046) • Peak pressures > 200 kPa less frequently found in indoor footwear 3) Ulcer recurrence rate: 26%
López-Moral et al. (2019) [31]	RCT Specialized diabetic foot unit, Spain	18 + years old Diabetes (T1/T2) LOPS due to PN Previous foot ulcer under metatarsal head	51 diabetic patients Control: 25 Intervention: 26	• Intervention: Male (n (%)): 24 (92%) Female (n (%)): 2 (8%) T1DM (n (%)): 0 (0%) T2DM (n (%)): 26 (100%) Age (yrs): 61 ± 8.1 BMI (kg/m2): 28.71 ± 4.97 Diabetes duration (yrs): 14 ± 8.4 • Control: Male (n (%)): 23 (92%) Female (n (%)): 2 (8%) T1DM (n (%)): 1 (4%) T2DM (n (%)): 24 (96%) Age (years): 60 ± 8.6 BMI (kg/m^2^): 30.07 ± 4.24 Diabetes duration (yrs): 17 ± 10.0	Therapeutic footwear with a rigid rocker sole Follow-up: 6 months OR development of recurrence event	• Ulcer recurrence • Footwear adherence • Loss of protective sensation • Forefoot deformities • Range of joint mobility • Ankle dorsiflexion	1) Ulcer recurrence: • Control: 16 (64%) • Intervention: 6 (23%) • RRR: 64%, NNT: 2.43 2) Ulcer recurrence (≥ 60% adherence): • Control: 14 (78%) • Intervention: 4 (22%) • RRR: not given 3) Ulcer-free survival time (intervention): • *p* = 0.019 (0.086–0.807) HR: 0.263
Abbott et al. (2019) [30]	RCT Two multidisciplinary outpatient diabetic foot clinics in the United Kingdom	≥ 18 years old Diabetes (T1/T2) Previous ulceration on weight-bearing surfaces Presence of peripheral neuropathy Able to walk independently for 30 steps	58 diabetic persons with PN Control: 26 Intervention: 32	• Intervention: Age (years): 59.1 ± 8.5 Sex (m/f): 28/4 T2DM: 23 Diabetes duration (yrs): 22.2 ± 14.3 BMI (kg/m^2^): 31.9 ± 5.8 Abnormal SW filament (L/R): 29/31 • Control: Age (years): 67.1 ± 9.6 Sex (m/f): 23/3 T2DM: 22 Diabetes duration (yrs): 21.2 ± 10.7 BMI (kg/m^2^): 30.9 ± 4.8 Abnormal SW filament (L/R): 19/17	Audiovisual alerts from in-sole plantar pressure system Follow up: 18 months	• Ulcer recurrence • Device adherence • Adverse event data • Time to ulceration • Callus severity	1)Ulcer recurrence (18 months): • Control: 6 • Intervention: 4 • Significance: *p* = 0.29 2) Ulcer recurrence (device adherent subgroup analysis): • 86% risk reduction in re-ulceration rate vs. control • IRR: 0·14 (0·03–0·63) *p* = 0·011 3) Adherence rates (device data; Median): • Control: 6.9 h/day (4.5–8.9) • Intervention: 6.1 h/day (4.3–7.3) • Significance: *p* = 0.22 4) Adherence rates (self-reported; Median): • Control: 4.3 h/day (2.9–5.8) • Intervention: 5.2 h/day (2.9–7.7) • Significance: *p* = 56) 5) Adverse events relating to lower limb (total) • Control: 29 • Intervention: 22
Piaggesi et al. (2016) [34]	Randomised multicentre prospective study Five specialised diabetic foot clinics in Italy	Diabetic (T1/T2) Diabetes duration of ≥ 5 years Present ulceration (Grade IA/IIA, UTDWC) for ~ 6 weeks ABP index ≥ 0.9 with 2 palpable pulses in affected foot	60 diabetic persons Group A: 20 Group B: 20 Group C: 20	• Group A: Females (%): 40% Age (years): 61.4 ± 9.7 Duration of diabetes (years): 9.8 ± 6.7 BMI (kg/m^2^): 30.2 ± 3.9 IA/IIA ulcers: 14/6 Previous ulceration (%): 50% • Group B: Females (%): 35% Age (years): 59.8 ± 7.9 Duration of diabetes (years): 11.4 ± 7.3 BMI (kg/m^2^): 32.6 ± 4.1 IA/IIA ulcers: 15/5 Previous ulceration (%): 45% • Group C: Females (%): 45% Age (years): 62.3 ± 9.2 Duration of diabetes (years): 12.2 ± 8.4 BMI (kg/m^2^): 29.7 ± 3.3 IA/IIA ulcers: 16/4 Previous ulceration (%): 55%	Group A: Total contact cast (TCC) Group B: Walking boot rendered irremovable (i-RWD) Group C: Removable walking boot (RWD) Follow-up: 90 days OR complete re-epithelialization of ulcer	• Ulcer healing time • Ulcer size reduction • Number of adverse events • Patient acceptance • Cost of devices	1) Cost of devices per patient • TCC: €457.8 • i-RWD: €163.4 • RWD: €144.0 2) Cost per days of treatment: • TCC: €242.8 ± 69.3 • i-RWD: €82.5 ± 33.7 • RWD: €67.9 ± 39.4 3) Total cost of devices: • TCC: €9156.7 • i-RWD: €3268.2 • RWD: €2880.4 4) Adverse events: • Group A: 7 patients (35%) • Group B: 2 patients (10%) • Group C: 1 patient (5%) • Group A vs. Group C: *p* = .0436 • No SAE reported
Craig et al. (2013) [32]	Economic evaluation Outpatients and community settings within NHS Borders in Scotland	Diabetic (T1/T2) Previous or active ulceration Risk factors for DFU, incl. LOPS	Audit: 19 patients Ulcer preventive pathway: 508 Ulcer curative pathway: 178	Unavailable	Soft-heel casting Follow up: 12 months	• Costs of healed and unhealed ulcers per patient • Resource costs • Ulcer healing rates	1) Cost per prevention pathway patient (no new ulcers): soft-heel cast cost less per patient (£489) than orthotic footwear: (£558) 2) Cost per prevention pathway patient (new ulcer develops): soft-heel cast cost less per patient (£6,359) than orthotic footwear (£6,726) 3) Cost per curative pathway patient (inpatients): soft-heel cast cost less per patient (£6,991) than orthotic footwear (£7,540) 4) Cost per curative pathway (outpatients) cost less per patient (£5,359) than orthotic footwear (£5,977) Soft-heel cast: £5,359 5) Resource cost: Soft-heel casting cost per patient estimated at £29, with £11 for materials, £16.60 for staff time and mean of 1.05 cast per person Orthotic boot cost per patient estimated at £90, with £90 for materials and £8 for staff time 6) Ulcer healing (total): Soft-heel casting: 42/65 (64%) Orthotic footwear: 47/91 (52%)
Bus et al. (2013) [28]	RCT Multidisciplinary outpatient diabetic foot clinics of two academic and eight large general public hospitals across the Netherlands	≥ 18 years old Diabetic (T1/T2) LOPS due to PN Healed plantar foot ulcer 18 months prior to randomisation New prescription of custom-made footwear	171 diabetic patients Control: 86 Intervention: 85 Subgroup analysis (Pts with ≥ 80% adherence across both groups: 79)	• Intervention Age (years): 62.6 ± 10.6 Male sex: 82.3% T2DM: 67.1% Diabetes duration (yrs): 19.9 ± 15.1 BMI (kg/m^2^): 30.9 ± 6.4 LOPS (abnormal SW monofilament): 94.1% • Control: Age (years): 63.9 ± 10.1 Male sex: 82.6% T2DM: 75.6% Diabetes duration: 14.7 ± 11.2 BMI (kg/m^2^): 30.4 ± 4.9 LOPS (abnormal SW monofilament): 91.9%	Custom-made footwear with improved offloading properties Follow-up: 18 months	• Ulcer recurrence • Ulcer recurrence in subgroup with ≥ 80% adherence • In-shoe peak pressure	1) Ulcer recurrence (all patients): • Intervention: 38.8% (n = 33) • Control: 44.2% (n = 38) • Relative risk (RR) reduction: 11%, OR 0.80 [95% CI 0.44–1.47], p = 0.48 2) Ulcer recurrence (adherence ≥ 80%): • Intervention: 25.7% (n = 9) • Control: 47.8% (n = 21) • RR reduction: 46%, OR 0.38 [95% CI 0.15–0.99], P = 0.045 3) In-shoe peak pressure at follow-up (post-modification; all regions > 200 kPa): • Intervention: 221 ± 51 • Control: 274 ± 66 • Effect: β -53 (-65 to -42) 4) In-shoe peak pressure at follow-up (post-modification; previous ulcer locations > 200 kPa): • Intervention: 200 ± 47 • Control: 304 ± 101 • Effect: β −69 (−89 to −49) 5) In-shoe peak pressure at follow-up (post-modification; previous ulcer locations < 200 kPa): • Intervention: 127 ± 44 • Control: 133 ± 42 • Effect: β −6 (−14 to 2)
Paton et al. (2012) [21]	RCT Two centres in South West England	Diabetic (T1/T2) Confirmed insensate or diminished sensation Confirmed palpable or biphasic pulses No ulceration in last ≥ 6 months Scored Grade 0 on Wagner classification Able to walk minimum 10 m unaided	119 diabetic persons at baseline Control: 59 Intervention: 60 104 diabetic persons at 6-months endpoint Control: 57 Intervention: 53	• Intervention: Age (years): 71 ± 10 BMI (kg/m^2^): 32 ± 10 Gender (m/f): 48/12 Diabetes type (T1/T2/unknown): 2/57/1 Neuropathy: 60 Diabetes duration (years): 9 ± 9 • Control: Age: 70 ± 10 BMI (kg/m^2^): 32 ± 11 Gender (m/f): 42/17 Diabetes type (T1/T2/unknown): 2/57/0 Neuropathy: 59 Diabetes duration (yrs): 9 ± 8	Custom-made functional insole Follow-up: 6 months	• Peak pressure, forefoot pressure time integral, total contact area, forefoot rate of load, duration of load as a percentage of stance • Patient perceived foot health (Bristol Foot Score), quality of life (Audit of Diabetes Dependent Quality of Life) • Cost of supply and fitting	1) Peak pressure reduction (baseline): • Intervention (Baseline): 189 kPa (39%) • Control (Baseline): 199 kPa (35%) 2) Peak pressure reduction (6 months): • Intervention (6 months): 239 kPa (37%) • Control (6 months): 187 kPa (31%) • Significance: *F* = 2.687, Eta^2^ = 0.023 3) Mean costs (insoles only): • Intervention: £137.65 • Control: £31.73 4) Mean costs (total): • Intervention: £656.03 • Control: £554.28 • Significance: *t* = − 8.942, *p* < 0.001 5) Bristol Foot Score (baseline): • Intervention: 44 • Control: 42 6) Bristol Foot Score (6 months): • Intervention: 41 • Control: 40 • Significance: *F* = 0.449, Eta^2^ = 0.004, *p* = 0.481 7) Audit of Diabetes Dependent Quality of Life (baseline): • Intervention: −2.2 • Control: −2.39 8) Audit of Diabetes Dependent Quality of Life (6 months): • Intervention: −2.54 • Control: −2.39 • Significance: *F* = 0.002, Eta^2^ = 0.000, *p* = 0.963
Rizzo et al. (2012) [29]	Prospective RCT Diabetic foot unit at University of Pisa, Italy	≥ 18 years old Diabetes (T1/T2) Diabetes duration of ≥ 5 years Ulcerative risk score of ≥ 2	298 diabetic persons Control: 150 Intervention: 148	• Intervention: Diabetes type (T1/T2): 21/127 Age (yrs): 68.1 ± 14.1 Diabetes duration (yrs): 18.1 ± 12.1 • Control: Diabetes type (T1/T2): 27/123 Age (yrs): 66.2 ± 9.4 Diabetes duration (yrs): 17.4 ± 10.9	Custom-made orthoses and shoes Follow-up: 1, 3 & 5 years	• Ulcer incidence • Costs	1) Ulcer incidence (12 months): • Group A (Control): 38.6% (*n* = 58) • Group B (Intervention): 12.8% (*n* = 17) • Significance: *χ*^2^ = 19.187, *p* < 0.0001 2) Ulcer incidence (3 yrs): • Group A (Control): 61.0% • Group B (Intervention): 17.6% • Significance: *χ*^2^ = 38.686, P < 0.0001 3) Ulcer incidence (5 yrs): • Group A (Control): 72.0% • Group B (Intervention): 23.5% • Significance: *χ*^2^ = 46.154, P < 0.0001 4) Cost of orthosis and shoe manufacturing (12 months): • Total: €99,990 • Per Patient: €675

*LOPS* loss of protective sensation, *DFU* diabetic foot ulceration, *PN* peripheral neuropathy, *RRR* relative risk reduction, *NNT* number needed to treat, *HR* hazard ratio, *UTDWC* University of Texas Diabetic Wound Classification, *ABP* ankle-brachial pressure, *T1DM* type 1 diabetes mellitus, *T2DM* type 2 diabetes mellitus

^*^Due to variations in baseline characteristics reported, only data consistent between studies or relevant to the review are displayed in the table. Furthermore, data are reported in the order they appear within the selected studies

Four studies took place in the United Kingdom, with two conducted in England [21, 27], one in Scotland [32], and one not defined [30]. Two studies were conducted in Italy [29, 34], two took place in the Netherlands [28, 33] and a singular study took place in Spain [31].

Five studies were conducted in specialist diabetic foot clinics or units [29–31, 33, 34]. Collings et al. [27] and Paton et al. [21] defined their settings as centres across South West England, while Craig et al. [32] described their settings as community and outpatient units across NHS Borders, a health board within NHS Scotland.

The utility of study results depends on whether they can be generalised to the larger population, thus the samples of included studies should adequately represent the wider neuropathic diabetic population. Namely, they should be diabetic and diagnosed with peripheral neuropathy or another clinical pathology leading to sensory loss in the lower limbs. On this basis, external validity across studies was relatively good; of the pooled sample of 853 participants from across the included literature, 40.56% (n = 346) had peripheral neuropathy or significant loss of protective sensation in the lower limbs.

One study [29] used an ulcerative risk score in lieu of previous ulceration monitoring; meaning it did not technically meet the inclusion criteria. However, after debate within the review team it was decided that this paper could be included as the study population was similar to those of other studies, and the outcomes were broadly relevant.

### Methodological quality

The methodological quality of the selected studies (bar one study [33]) was considered high. Each study provided ample information on interventions, outcomes, and results; three studies also reported details of adverse events [27, 30, 34]. Comprehensive details regarding methodological quality can be found in Tables 4, 5, 6.

**Table 4:** Quality assessment table for RCT studies included in the synthesis. Questions are derived from the JBI Checklist for Randomized Controlled Trials

References	Q1	Q2	Q3	Q4	Q5	Q6	Q7	Q8	Q9	Q10	Q11	Q12	Q13
[27]	Y	Y	Y	Y	Y	Y	Y	Y	Y	Y	Y	Y	Y
[30]	Y	Y	Y	Y	Y	N	Y	Y	Y	Y	Y	Y	Y
[31]	Y	Y	Y	Y	Y	UC	Y	Y	Y	Y	Y	Y	Y
[34]	Y	UC	Y	Y	UC	Y	Y	Y	Y	Y	Y	Y	Y
[28]	Y	Y	Y	Y	Y	Y	Y	Y	Y	Y	Y	Y	Y
[21]	Y	Y	Y	Y	UC	Y	Y	Y	Y	Y	Y	Y	Y
[29]	Y	UC	Y	Y	UC	UC	Y	Y	Y	Y	Y	Y	Y

*Y* Yes, *N* No, *UC* Unclear

**Table 5:** Quality assessment table for the quasi-experimental study included in the review. Questions are derived from the JBI checklist for Quasi-experimental studies

Reference	Q1	Q2	Q3	Q4	Q5	Q6	Q7	Q8	Q9
[33]	Y	Y	N	N	Y	Y	UC	Y	Y

*Y* Yes, *N* No, *UC* Unclear

**Table 6:** Quality assessment table for the economic evaluation study included in the review. Questions are derived from the JBI checklist for economic evaluations

Reference	Q1	Q2	Q3	Q4	Q5	Q6	Q7	Q8	Q9	Q10	Q11
[32]	Y	Y	Y	UC	Y	Y	UC	Y	Y	Y	Y

*Y* Yes, *N* No, *UC* Unclear

Blinding protocols varied across the included studies. Double-blinding (i.e. blinding of participants and treatment providers) occurred in four of the RCTs [27, 28, 30, 31]. It was unclear in two studies whether there was blinding of treatment providers [21, 34], and it was unclear whether there was participant blinding in one study [34]. Blinding was not present in three studies [29, 32, 33]. Triple-blinding (i.e. blinding of participants, healthcare providers, and outcome assessors) was present in two of the RCTs [27, 28]. Five studies used randomised allocation of intervention with concealment [21, 27, 28, 30, 31]; it was unclear whether concealment was used in one randomised study [34].

### Results synthesis

#### Interventions & comparators

Four studies used footwear as interventions [28, 29, 31, 33]; of these, three used custom-made footwear [28, 29, 33] and one employed therapeutic footwear with a rigid rocker sole [31]. Comparisons across the studies employing custom-made footwear were heterogenous: one study [28] compared custom-made footwear with improved offloading properties to non-improved custom-made footwear; one [29] compared custom-made footwear to standard treatment; one [31] compared therapeutic footwear with rigid rocker soles to therapeutic footwear with semi-rigid soles [31]; and one [33] lacked a control group, as all participants had custom-made footwear.

Three studies used custom-made insoles as interventions [21, 27, 30]; of these two studies [21, 27] compared custom-made insoles with prefabricated insoles, while one study [30] provided an intelligent insole system to all participants. In this study [30] the intervention group received audio-visual alerts from said system when aberrant pressure was detected, while the control group did not. Of the remaining two studies, one [32] employed soft-heel casting and compared it to standard therapeutic footwear, and one [34] compared three different orthotic interventions: total contact casts, walking boots rendered irremovable, and removable walking boots.

#### Prevention of ulcer incidence/recurrence

The primary outcome of interest (i.e. efficacy of custom-made offloading device in terms of preventing ulcer incidence or recurrence), was investigated by six studies [27, 29–33]. Abbott et al. [30] tested the effectiveness of an intelligent insole system in reducing ulcer recurrence in diabetic patients with previous ulceration and peripheral neuropathy over 18-months. At 18-months, a total of 10 ulcers (17%) recurred across both groups, with six recurring in the control group and four in the intervention group. Poisson regression demonstrated a 71% risk reduction of re-ulceration in the intervention group (IRR 0·29; 95% CI 0·09–0·93; p = 0.037). Ulcer recurrence further decreased by 86% within the intervention group in a subgroup analysis of system-adherent patients (i.e. wearing ≥ 4.5 h a day; IRR 0·14, 95% confidence interval [CI] 0·03–0·63; p = 0·011).

Collings et al. [27] compared foot ulcer incidence in two diabetic neuropathy groups over a period of 12-months. The intervention group were provided with instant optimised insoles accommodating for areas of ulceration. At the 12-month follow-up, foot ulcer incidence was lower in the intervention group (22.5%) compared to the control group (33.3%), although statistical outcomes were not presented.

Rizzo et al. [29] assessed the impact of custom-made orthoses/shoes and a structured prevention program on DFU incidence in diabetic patients at high risk. Ulcer incidence was measured at 1-, 3-, and 5-year follow-ups. Both the intervention and control groups were enrolled in the prevention programme; the intervention group were provided with custom-made orthoses and shoes. At 12-months follow-up, ulcer incidence was significantly lower in the intervention group (12.8%) compared to the control group (38.6%; *χ*^2^ = 19.187, *p* < 0.0001). This difference was still significant at the 3-year (17.6% vs. 61.0%; *χ*^2^ = 38.686, *p* < 0.0001) and 5-year follow-ups (23.5% vs. 72.0%; *χ*^2^ = 46.154, *p* < 0.0001). The separate effects of the custom-made orthoses/shoes and the structured prevention program could not be estimated from the available data.

López-Moral et al. [31] analysed the efficacy of therapeutic footwear with a rigid rocker sole in preventing ulcer recurrence among diabetic patients with peripheral neuropathy. The intervention group were provided with therapeutic footwear with rigid rocker sole, while the control group were given therapeutic footwear with semi-rigid soles. Ulcer recurrence was lower in the rigid rocker sole group (23%) versus the semi-rigid sole group (64%). Survival analysis on a group of patients with ≥ 60% adherence to therapeutic footwear revealed that rigid rocker sole interventions significantly lessened ulcer development in patients with peripheral neuropathy and histories of DFU (*p* = 0.019; 95% CI 0.086–0.807; hazard ratio: 0.263).

Two studies briefly examined ulcer incidence/recurrence [32, 33]. In their study, Keukenkamp et al. [33] measured the effects of custom-made indoor insoles and adherence on ulcer recurrence. Groups were determined by baseline indoor adherence (i.e. how often indoor insoles were worn, expressed as percentages of time). The primary group had low indoor adherence (< 80%), while the secondary group had high indoor adherence (≥ 80%). Custom-made indoor footwear was provided to both groups. In the high indoor adherence (≥ 80%) group, ulcer recurrence was low, with 26% of participants developing foot ulcers; further statistical findings were not reported. Craig et al. [32] examined the effectiveness of soft-heel casting (semi-rigid tape placed over primary dressings and fastened by secondary dressings) in ulcer incidence reduction using audits, previously published data, and expert opinion. Two groups were modelled: a preventative group (n = 508) with a high-risk of developing ulceration; and a curative group (n = 178) with an estimated ulceration rate of 3.5% annually. Ulcer healing was higher amongst soft-heel casting users (64%) compared to orthotic footwear users (52%); however further statistical evidence was not reported.

Only one study found no significant result after follow-up [28]. Bus et al. [28] examined the effect of custom-made footwear with improved offloading on plantar ulcer recurrence in neuropathic diabetic patients with previous ulceration. The intervention group were given improved, custom-made footwear with ~ 20% peak pressure relief. At 18-months follow-up, ulcer recurrence did not significantly differ between the two groups (intervention: 38.8%; control: 44.2%; relative risk reduction: 11%; odds ratio [OR] 0.80; 95% CI 0.44–1.47, *p* = 0.48). However, a subgroup analysis of patients with ≥ 80% adherence did show significant difference in ulcer recurrence between the two groups (intervention: 25.7%; control: 47.8%; relative risk reduction: 46%; OR 0.38; 95% CI 0.15–0.99, *p* = 0.045).

#### Peak pressure reduction

Four studies evaluated the effects of custom offloading devices on peak plantar pressure reduction [21, 27, 28, 33]; plantar pressure being the pressure between the foot and the support surface (e.g. shoe) during everyday movement. Two studies described positive outcomes in peak pressure reduction. At 6-months follow-up, Collings et al. [27] found that optimized insoles reduced mean peak plantar pressures in both regions of interest, defined by the authors as recently healed ulcer sites or callus/corns, or areas with mean peak plantar pressures greater than 350 kPa (Region of Interest [RoI]1: *M* = 639.8 kPa, *SD* = 332.3 kPa; RoI 2: *M* = 717.0 kPa, *SD* = 476.6 kPa). Follow-up at 12-months also favoured the intervention; however, as the authors noted concerns over prolonged pressure reduction with the insoles due to uncertainty over their durability beyond 6 months. The custom-made footwear with improved offloading used by Bus et al. [28] led to significantly lower in-shoe peak pressures in the intervention group (221 ± 51 kPa) than the control group (274 ± 66 kPa) across all regions of interest (β −53 (−65 to −42); p < 0.001).

The two other studies [21, 33], however, found no reduction in peak pressure. Paton et al. [21] used custom-made functional insoles as an intervention to reduce ulceration risk factors in diabetic patients with peripheral neuropathy, including peak pressure reduction. Overall, no significant differences were found between the two groups in terms of peak pressure reduction at 6-months follow-up (intervention: 239 kPa (37%); control 187 kPa (31%); F = 2.687, Eta2 = 0.023, p = 0.104). Similarly, Keukenkamp et al. [33] found no significant differences between custom-made and regular indoor footwear in peak plantar pressure reduction, though peak pressure measurements of > 200 kPa occurred less frequently with the custom-made footwear.

#### Costs

Four studies analysed the costs of custom-made offloading devices [21, 29, 32, 34]. Methods for cost measurements were heterogenous across studies. Craig et al. [32] estimated the costs of using soft-heel casting versus standard orthotic boots per patient and per patient pathway in GBP (£). Overall, soft-heel casting was less expensive per patient compared to standard orthotic boots. In the preventative group, soft-heel casting costs per patient were lower than orthotic footwear cost per patients in both subgroups (no new ulcers: soft-heel cast = £489; orthotic footwear = £558; new ulcers: soft-heel cast = £6,359; orthotic footwear = £6,726). For the curative group, soft-heel casting costs per patient were also lower than orthotic footwear for inpatients (soft-heel cast = £6,991; orthotic footwear = £7,540) and outpatients (soft-heel cast = £5,359; orthotic footwear = £5,977). Costs per individual patient was £29 for soft-heel casting, with £11 spent on materials, £16.60 for staff time to prepare and fit casts, and a mean of 1.05 casts per person. Comparably, it costs £98 for orthotic footwear, with the boot itself costing £90 in addition to fitting by a podiatrist at £8 per patient.

Piaggesi et al. [34] tested the effectiveness of three walking boots in diabetic patients with DFU staged at IA (i.e. superficial wounds) or IIA (i.e. wounds penetrating to tendons or capsules), according to the University of Texas Diabetic Wound Classification [35]. Patients were randomised into three groups: group A were provided with total contact casts; group B received walking boots rendered irremovable; and group C were given removable walking boots. Total contact casts were found to be the most expensive device, costing approximately €457.80 per patient; comparatively, irremovable and removable walking boots were less costly, costing around €163 and €144 per patient, respectively.

In one study [21] the total mean cost of the intervention was significantly more (£656.03) than the control (£554.28; t = −8.942, p < 0.001). Mean cost of the customised insole alone was £137.65, while the mean cost of the standard insole alone was £31.73. Rizzo et al. [29] only measured the cost of the custom devices for the intervention group at 12-month follow-up. Costs for manufacturing orthoses and shoes was estimated to be €675 per patient; no further information on costs or costs breakdown were available.

#### Device adherence

Five studies measured device adherence [27, 28, 30, 31, 33]. Collings et al. [27] recorded insole adherence in both groups using an integrated temperature sensor; high adherence was considered to be wearing the insoles for > 8 h of daylight, as defined by Waaijman et al. [36]. Complete wear data from 44 participants across both groups were uploaded and analysed; 45.5% (n = 20) wore insoles for < 4 h per day, 38.6% (n = 17) wore insoles for 4 to 8 h, and 15.9% (n = 7) for > 8 h per day.

Keukenkamp et al. [33] primarily focused on adherence to custom-made indoor insoles in their study, using a temperature-based sensor to measure daily step counts and footwear use over seven consecutive days. Low adherence was defined as < 80% of total indoor steps in prescribed footwear; conversely, high adherence was defined as ≥ 80% of the same measurement. At baseline, 23 participants had low adherence; following provision of custom-made indoor footwear, average indoor adherence increased from 48 to 71% at 1-month follow-up (*p* = 0.001, *r* = 0.74) and to 77% at 12-months follow-up (*p* < 0.001; *r* = 0.78). Overall adherence, including outdoor use, significantly increased from baseline (65%) at 1-month (77%; *p* = 0.002; *r* = 0.66) and 12 months follow-up (87%; *p* < 0.001; *r* = 0.74). The length of time wearing custom-made footwear, both indoor and regular (i.e. footwear worn outdoors), also increased from 8.6 h/day to 9.3 h/day (*p* = 0.0014; *r* = 0.68) and 12.0 h/day (*p* = 0.002; *r* = 0.75), respectively.

Abbott et al. [30] measured adherence to an innovative insole system in both intervention and control groups using device-recorded data and patient questionnaires. Device-recorded data showed no significant difference in median device usage time between control (6.9 h/day; interquartile range [IQR] 4.5–8.9) and intervention (median 6.1.h/day; IQR 4.3–7.6; *p* = 0.22). Similarly, there were no significant differences in self-reported median device usage time between control (4.3 h/day; IQR 2.9–5.8) and intervention (5.2 h/day; IQR 2.9–7.7; *p* = 0.56).

Two studies briefly measured adherence in order to conduct statistical analyses on other outcomes. Both López-Moral et al. [31] and Bus et al. [28] recorded adherence to perform subgroup analyses of ulcer recurrence in patients with high adherence rates; the former used ≥ 60% as a cut-off value for adherence, while the latter used ≥ 80%. López-Moral et al. [31] used questionnaires distributed at consultations to measure device adherence; 46 participants (90.2%) were regarded as highly adherent. Bus et al. [28] calculated adherence from temperature-based monitors inserted in the custom-made footwear and determined 79 of the 171 participants (46%) were highly adherent.

## Discussion

Overall, considering the evidence available, we can conclude custom-made offloading devices may be an effective intervention for preventing ulcer recurrence/incidence in diabetic patients with peripheral neuropathy. However, we are unable to confirm whether variable offloading devices improve rates of adherence in users, nor are we able to determine the cost-effectiveness of these devices from the available evidence. Evidence of the effect of custom-made offloading devices on peak plantar pressure is also mixed.

The aetiology of DFU is multifactorial, and while peripheral neuropathy is most commonly associated with ulceration in diabetic populations, it is not the singular cause; peripheral artery disease is another causative factor that results in the formation of ischaemic ulcers in affected patients [37, 38]. While this systematic review focuses on the effectiveness of custom-made/variable offloading devices on foot ulceration in diabetic populations generally, almost half of the identified studies included peripheral neuropathy or lower limb sensory loss as a primary factor in DFU development, as indicated by Table 3. Therefore, the outcomes surmised here may be less applicable to ulceration caused by other conditions (such as peripheral artery disease). Clinical inference of these results should be limited to patient populations with diabetes and peripheral neuropathy.

Our primary aim, to determine the efficacy of custom-made offloading devices for preventing DFU incidence or recurrence, was achieved. In five of the studies analysed there were significant differences in ulcer incidence/recurrence between intervention groups and control groups. Only Bus et al. [28] found no such differences after follow-up; however, a subgroup analysis on patients with high adherence rates (i.e. ≥ 80%) did find significant reductions in ulcer incidence in the intervention group versus the control group. Furthermore, ulcer incidence or recurrence outcomes were reported relatively consistently as percentages, except for López-Moral et al. [31] who performed a survival analysis on participants, thus reporting their result as a hazard ratio.

This efficacy may be attributable to different factors. For example, lower limb structure varies among people with diabetes, with ulceration and musculoskeletal deformities common findings in those with moderate to severe disease [39, 40]. Custom-made offloading devices can accommodate for these anatomical features by conforming to the foot as it presents in the clinic. Materials may also influence ulceration rates. In the construction of custom-made offloading devices, clinicians and orthotists can select materials that reduce or prevent risk factors specific to foot ulceration, as well as provide more comfort for patients.

We were unable to determine whether custom-made offloading devices were more cost-effective than standard offloading devices due to a lack of evidence. Overall the data reviewed were not comprehensive enough to form conclusions on cost-effectiveness. In particular there was a lack of information on specific costs, namely material costs, service costs, and long-term costs (i.e. costs at/after follow-up periods). Were they present, these costs would also have needed comparison to those of standard offloading devices. While costs per patient were described, this does not explain whether custom-made devices are more cost-effective than standard devices. We suggest that future studies comparing custom-made devices to standard ones should detail all costs involved, including manufacturing and service costs. Additionally, costs at follow-up periods and cost-effectiveness comparisons with standard insoles should also be examined.

We also cannot conclude whether adherence rates are higher in users of custom-made devices compared to those using standard devices, or if higher adherence leads to reductions in ulcer recurrence. These conclusions are based on two observations. Firstly, there were not enough studies comprehensively evaluating adherence as an outcome; only Keukenkamp et al. [33] measured adherence as a primary outcome. Conversely, López-Moral et al. [31] and Bus et al. [28] only briefly measured adherence for the purpose of additional sub-group statistical analyses. Secondly, methods used to measure adherence were heterogenous. For example, Collings et al. [27] and Keukenkamp et al. [33], each used integrated temperature sensors, but how they calculated adherence differed greatly; the former used a specific length of time (i.e. > 8 h/day) to determine high adherence, while the latter used daily step counts over seven days to establish high and low adherence rates. This variance limits the generalisability of the results. Given that adherence to treatment is an issue across healthcare [41, 42], there should be an effort towards developing a standardised method of measuring adherence that can then be used in future studies.

Finally, we were unable to determine the effectiveness of custom-made offloading devices for reducing peak pressure reduction. There were not enough data, and the data extracted were too mixed to form a definitive conclusion. Pressure is a major risk factor in the development and progression of DFU, so it should be accounted for when providing diabetic patients with footwear and other orthoses. However, we should note that during our initial screening of studies, there were numerous studies focusing on peak pressure reduction in custom-made offloading devices as a primary outcome; these were not included in our study as peak pressure reduction was only considered a secondary aim. Thus we believe there is potential for a separate review focussing on the effects of custom-made offloading devices on peak pressure reduction in diabetic patients. Otherwise, we recommend that future trials and other experimental studies incorporate peak pressure reduction as an outcome alongside ulcer incidence/recurrence.

It should be noted that there are some previously published systematic reviews examining the use of offloading devices on DFU [43–45]. However, we believe our work differs significantly from these studies by focusing primarily on custom-made offloading devices and their effects on the whole diabetic foot, as opposed to all offloading devices or specific anatomical areas. Additionally, we also examine the economic costs associated with the use of custom-made devices, an aspect of DFU management which is less studied compared to ulcer recurrence or plantar pressure despite its importance in determining the clinical utility of an intervention. Our work also accounts for more recent progress in custom-made offloading devices, so the data analysed are the latest available. Our decision not to perform a meta-analysis was influenced primarily by the lack of data currently available and the heterogeneity of data, thus we deemed a narrative synthesis to be sufficient for this study.

## Conclusions

In conclusion, we believe custom-made offloading devices can be effective in preventing ulcer incidence/recurrence in diabetic patients with or at risk of developing DFU, compared to standard offloading devices. However, we cannot declare whether these custom-made devices are cost-effective, nor can we determine whether patients are more likely to adhere to them and whether they are effective in reducing peak pressures.

Cost-effectiveness is perhaps the most important factor preventing full recommendation of custom-made offloading devices. Future research comparing such devices versus standard devices should include all costs involved in their production, including costs for staff time and long-term costs of device maintenance. Additionally, use of a standardised method for measuring adherence in this context would be beneficial, as well as including a standardised measure of peak pressure as a primary outcome alongside ulcer incidence/reduction.

Despite these caveats, there is clearly a growing body of evidence which demonstrates the clinical benefits of custom-made offloading devices for preventing DFU.

## Data Availability

All data reported in this paper are available from the original sources, please see reference list and Table 3 for further details.

## Abbreviations

DFU: Diabetic foot ulceration
QALY: Quality-adjusted life years
IRR: Incidence risk ratios
RCT: Randomized controlled trial
CI: Confidence interval
OR: Odds ratio
LOPS: Loss of protective sensation
PN: Peripheral neuropathy
RRR: Relative risk reduction
NNT: Number needed to treat
HR: Hazard ratio
UTDWC: University of Texas Diabetic Wound Classification
ABP: Ankle-brachial pressure
T1DM: Type 1 diabetes mellitus
T2DM: Type 2 diabetes mellitus
RoI: Region of interest
IQR: Interquartile range
PPIE: Patient and Public Involvement and Engagement
NIHR: National Institute for Health and Care Research
